# A phase 2b/3b MenACWY-TT study of long-term antibody persistence after primary vaccination and immunogenicity and safety of a booster dose in individuals aged 11 through 55 years

**DOI:** 10.1186/s12879-020-05104-5

**Published:** 2020-06-18

**Authors:** Charissa Fay Corazon Borja-Tabora, Paula Peyrani, Chris Webber, Marie Van der Wielen, Brigitte Cheuvart, Nathalie De Schrevel, Veronique Bianco, Emmanuel Aris, Mark Cutler, Ping Li, John L. Perez

**Affiliations:** 1 Research Institute for Tropical Medicine, Alabang Muntinlupa City, Metro Manila Philippines; 2 Pfizer Vaccine Clinical Research and Development, Collegeville, PA USA; 3 Pfizer Vaccine Clinical Research and Development, Hurley, UK; 4 Global Vaccines Research and Development, GlaxoSmithKline, Wavre, Belgium; 5 Global Vaccines Research and Development, GlaxoSmithKline, Rixensart, Belgium; 6 Global Vaccines Research and Development, GlaxoSmithKline, Rockville, MD USA; 7 Value Evidence, Medical, Research and Development, GlaxoSmithKline, Wavre, Belgium; 8 Pfizer Vaccine Research and Development, Pearl River, NY USA

**Keywords:** Antibody, Immunogenicity, MenACWY-TT, Meningococcal, Persistence, Booster, Vaccine

## Abstract

**Background:**

A previous phase 2 study demonstrated the immunogenicity of a single dose of meningococcal A, C, W, Y-tetanus toxoid conjugate (MenACWY-TT) or polysaccharide (MenACWY-PS) vaccine for up to 5 years in individuals aged 11–55 years. This follow-up study evaluated long-term antibody persistence up to 10 years and the immunogenicity and safety of a single MenACWY-TT booster dose given 10 years after primary vaccination.

**Methods:**

Blood draws were conducted annually in Years 7–10. At Year 10, all subjects received a MenACWY-TT booster dose. Blood was drawn at 1 month and safety data were collected ≤6 months postbooster. Study endpoints included immunogenicity during the persistence phase (primary), and immunogenicity and safety during the booster phase (secondary). Statistical analyses were descriptive.

**Results:**

A total of 311 subjects were enrolled in the persistence phase (MenACWY-TT, 235; MenACWY-PS, 76); 220 were enrolled in the booster phase (MenACWY-TT, 164; MenACWY-PS, 56). Descriptive analyses indicated that at Years 7–10, the percentages of subjects achieving serum bactericidal antibody assay using baby rabbit complement (rSBA) titers ≥1:8 and ≥1:128 were higher for serogroups A, W, and Y in the MenACWY-TT versus MenACWY-PS group; percentages were similar across groups for serogroup C. rSBA geometric mean titers (GMTs) for serogroups A, W, and Y were higher in the MenACWY-TT group and slightly higher in the MenACWY-PS group for serogroup C. One month postbooster, all primary MenACWY-TT and ≥98.1% of primary MenACWY-PS recipients had rSBA titers ≥1:8. For all serogroups, rSBA GMTs postbooster were higher in the MenACWY-TT versus MenACWY-PS group. Most local and general reactogenicity events were similar between groups and mild to moderate in severity. Adverse events at 1 month postbooster were 9.1% for the MenACWY-TT and 3.6% for the MenACWY-PS groups; all were nonserious.

**Conclusions:**

Immune responses to a single MenACWY-TT primary dose administered at age 11–55 years persisted in >70% of individuals evaluated at Years 7–10. A MenACWY-TT booster dose administered at Year 10 was safe and immunogenic with no new safety signals observed. These results provide important insights regarding long-term protection from primary vaccination and the benefits of booster dosing.

**Trial registration:**

Clinicaltrials.gov, NCT01934140. Registered September 2013.

## Background

Invasive meningococcal disease (IMD), including bacterial meningitis and septicemia, is caused by *Neisseria meningitidis* [1, 2]. Current mortality rates for untreated bacterial meningitis are nearly 50%, and patients who survive often experience permanent and disabling sequelae [1, 3]. The risk of IMD is highest in children younger than 2 years and in older adolescents and young adults [1]. Of the 12 *N meningitidis* serogroups identified to date, 5 (A, B, C, W, Y) cause most cases of disease [1, 3, 4].

Since the introduction of the first polysaccharide meningococcal vaccines in the 1970s, new formulations with improved immunogenicity have become available [5, 6]. Specifically, meningococcal polysaccharide conjugate vaccines use carrier proteins, such as diphtheria toxoid (DT), tetanus toxoid (TT), and the diphtheria mutant toxin CRM_197_, which are covalently linked to the bacterial polysaccharides targeting serogroups A, C, W, and Y [1, 6]. These vaccines elicit immunologic memory, providing extended periods of protection compared with early vaccines [5]. However, despite predictions that meningococcal conjugate vaccines would provide protection for up to a decade after the primary vaccination, the duration of antibody persistence and its implications for the timing and impact of booster vaccination remain unclear [5, 7]. Retrospective studies show that circulating antibodies decrease within 3 to 8 years after a single dose of the quadrivalent meningococcal conjugate vaccines MenACWY-DT (Menactra®, Sanofi Pasteur Inc., Swiftwater, PA, USA) and MenACWY-CRM_197_ (Menveo®, GlaxoSmithKline Vaccines, Sovicille, Italy) [5, 7].

MenACWY-TT (Nimenrix®, Pfizer Ltd, Sandwich, UK) is a quadrivalent conjugate vaccine licensed for individuals aged ≥6 weeks to prevent meningococcal disease caused by serogroups A, C, W, and Y [8]. In a longitudinal phase 2b, open-label, randomized, controlled study (NCT00356369), the immunogenicity and antibody persistence through 5 years after a single dose of MenACWY-TT were compared with those of a single dose of a MenACWY polysaccharide vaccine (Mencevax™; MenACWY-PS, GlaxoSmithKline, Rixensart, Belgium) in healthy individuals aged 11 through 55 years [9, 10]. Results from serum bactericidal antibody assays using baby rabbit complement (rSBAs) showed that ≥72% of subjects vaccinated with MenACWY-TT had protective titers (≥1:8) at Year 5 [9, 10].

The objective of the current study was to evaluate the long-term antibody persistence 6 to 10 years after the primary vaccination in subjects in the primary MenACWY-TT versus MenACWY-PS study [10]. A secondary objective was to assess the immunogenicity and safety of a single MenACWY-TT booster given 10 years after the primary vaccination.

## Methods

### Study design and subjects

The designs of the primary vaccination study and the 5-year follow-up have been previously described [9, 10]. Briefly, healthy adolescents and adults from the Philippines and Saudi Arabia (aged 11–55 years at the time of the primary vaccination) were randomized 3:1 into 2 parallel groups to receive 1 dose of either MenACWY-TT or MenACWY-PS.

**Fig. 1:**
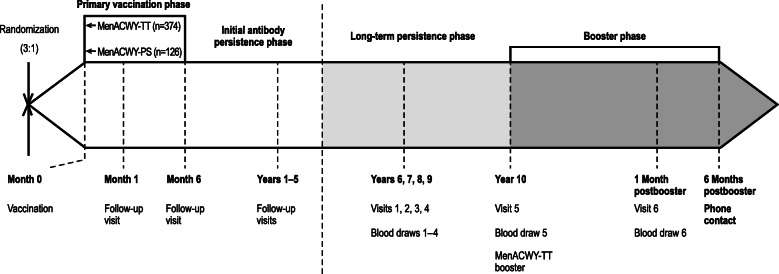
Study design. Randomization, the primary vaccination phase, the initial antibody persistence phase (Years 1–5), the current long-term persistence phase (Years 7–10)*, and the booster dosing phase are shown. *MenACWY* meningococcal A, C, W, Y, *PS* polysaccharide, *TT* tetanus toxoid *Study visits for Year 6 of the persistence phase were not conducted due to delay in study approval

The current long-term follow-up and booster study included subjects enrolled in the Philippines who were allowed to enter the study at any point up to the Year 10 visit. Target enrollment was 252 subjects in the MenACWY-TT group and 84 subjects in the MenACWY-PS group. The design of the entire study from the primary vaccination through the long-term persistence and booster phase is included as Fig. 1. No study visit occurred at Year 6 because approval from Food and Drug Administration Philippines was not obtained until the end of the Year 6 window.

Subjects eligible for inclusion in this study were aged 17 to 66 years at the time of entry; had completed the primary vaccination study; and were considered healthy on the basis of medical history and physical examination. The main exclusion criteria included previous meningococcal vaccination outside of the primary vaccination study; history of meningococcal disease due to serogroups A, C, W, or Y; immunosuppressive or immunodeficiency conditions; and chronic alcohol or substance abuse. Additional exclusion criteria applicable to administration of the booster dose at Year 10 included use of any investigational or nonregistered product other than the study vaccine within 30 days preceding the booster dose or planned use during the follow-up period; chronic administration of immunosuppressants or immune-modifying drugs within 6 months of booster administration; use of immunoglobulins or any blood products within 3 months of the booster dose; vaccination with tetanus toxoids within the preceding month; history of neurologic disorders, seizures, or Guillain-Barré syndrome; and acute disease or fever at the time of the booster vaccination.

### Vaccines

Subjects in both groups received a single intramuscular MenACWY-TT booster dose at Year 10 administered in the nondominant deltoid muscle.

### Study objectives

The primary immunogenicity objective was to evaluate the long-term antibody persistence (6–10 years after the primary vaccination) induced by MenACWY-TT compared with MenACWY-PS, assessed as the percentages of subjects with rSBA titers ≥1:8 and ≥1:128 against serogroups A, C, W, and Y and through comparison of rSBA geometric mean titers (GMTs). A secondary immunogenicity objective was to evaluate MenACWY-TT immunogenicity 1 month after the booster vaccination in terms of GMTs and the percentages of subjects with rSBA titers ≥1:8 and ≥1:128 against all 4 meningococcal serogroups. An additional secondary immunogenicity objective was to evaluate the percentage of subjects exhibiting a vaccine response 1 month postbooster; vaccine response was defined as an rSBA titer ≥1:32 in subjects who were seronegative (rSBA titer <1:8) before receiving the booster vaccination and as a ≥4-fold increase from prevaccination titers in subjects who were seropositive (rSBA titer ≥1:8) before vaccination. A third secondary objective was the assessment of safety after booster dosing, which encompassed the evaluation of reactogenicity and adverse events (AEs), as well as serious AEs (SAEs), new onset of chronic illnesses (NOCIs; eg, autoimmune disorders, asthma, type 1 diabetes, allergies), or occurrences of Guillain-Barré syndrome or meningococcal disease.

### Immunogenicity and safety assessments

Blood samples for immunogenicity analyses were collected from all subjects at 7, 8, 9, and 10 years after the primary vaccination and at 1 month after the booster vaccination. Functional antibody responses against serogroups A, C, W, and Y were assessed using rSBA assays performed at Public Health England (Manchester, UK). Samples were analyzed using the titers described above. Subjects or their parents or legal representatives were asked to record solicited local and general reactogenicity events through 3 days after receiving the booster vaccination (intensity scales for reactogenicity events are summarized in Additional File 1: Table S1). Nonreactogenicity AEs were evaluated through 31 days after the booster vaccination. SAEs, NOCIs, Guillain-Barré syndrome, and meningococcal disease were recorded from the booster vaccination until study end at 6 months postbooster.

### Statistical analysis

#### Analysis populations

For the analysis of persistence at Years 7, 8, 9, and 10, the total cohort at each year included all vaccinated subjects from the primary study who returned that corresponding year. The according-to-protocol (ATP) cohort for persistence was defined as all eligible subjects who received the primary vaccination with MenACWY-TT or MenACWY-PS; complied with blood sampling intervals as defined in the protocol; had not received immune-modifying drugs, investigational drugs, or other protocol-forbidden medications within the specified time frame; and had assay results for ≥1 tested antigen. Analyses for each of the persistence time points were performed using the respective ATP cohort for each year.

The booster total vaccinated cohort for immunogenicity included all subjects from the primary study who received a booster dose of the study vaccine and had data available for postbooster immunogenicity endpoint measures. The booster ATP cohort for immunogenicity included all subjects (1) who received a booster dose of the study vaccine, (2) who met previously described inclusion criteria, (3) who had no exclusion criteria, (4) whose administration site was known, (5) whose assay results for ≥1 tested antigen were available, and (6) who had not received a vaccine not foreseen by the study protocol before the postvaccination blood sample was drawn.

The booster total vaccinated cohort for safety included all subjects from the primary study with documented administration of the booster vaccine.

Analyses conducted for this study were descriptive in nature.

#### Antibody persistence

Analysis of antibody persistence was based on the ATP cohort for persistence for that year. For any year and vaccine group, if the percentage of subjects with serological results excluded from the ATP cohort was >5%, an analysis based on the total cohort for that year was also performed.

Two-sided 95% CIs were computed for immunogenicity analyses in each vaccine group. Exact 95% CIs for the percentage within a group were based on the method by Clopper and Pearson. The 95% CIs for GMTs were obtained within each vaccine group separately. For each GMT, the 95% CI for the mean of the log-transformed titer was first obtained assuming that log-transformed values were normally distributed with unknown variance. The 95% CI for the GMT was then obtained by exponential transformation of the 95% CI for the mean of log-transformed titer. Subgroup analyses of immunogenicity were conducted based on age at the time of vaccination (11–17 years and 18–55 years).

#### Immunogenicity modeling

To complement the descriptive analyses of observed persistence per time point and to minimize bias resulting from loss to follow-up after vaccination, a longitudinal analysis was performed for serogroups A, C, W, and Y at the last time point for rSBA titers, taking into account the age cohort. This analysis included all titers from the ATP cohort for each of Years 7, 8, 9, and 10. Further details regarding immunogenicity modeling methods are included in the Additional File 2: Supplementary Appendix.

#### Postbooster immunogenicity and safety

Analysis of postbooster immunogenicity was based on the booster ATP cohort for immunogenicity. For any vaccine group, if the percentage of subjects with serological results excluded from the booster ATP cohort was >5%, an analysis based on the total vaccinated cohort at 1 month postbooster was also performed. Postbooster immunogenicity was analyzed using data from the booster ATP cohort. For each sampling time point (Year 10 visit and 1 month postbooster), 95% CIs for GMTs and percentages of subjects with titers above the prespecified cutoffs were calculated using the same methods as for the persistence phase; 1 month postbooster, the percentages of subjects with a vaccine response and exact 95% CIs were also calculated. Subgroup analyses of postbooster immunogenicity were conducted based on age at the time of primary vaccination (11–17 years and 18–55 years).

The primary analysis of safety was performed on the booster total vaccinated cohort. Calculations of the percentages of subjects reporting local and general reactogenicity events, nonreactogenicity AEs and SAEs, and NOCIs were performed with exact 95% CIs.

## Results

### Subject disposition and demographics

**Fig. 2:**
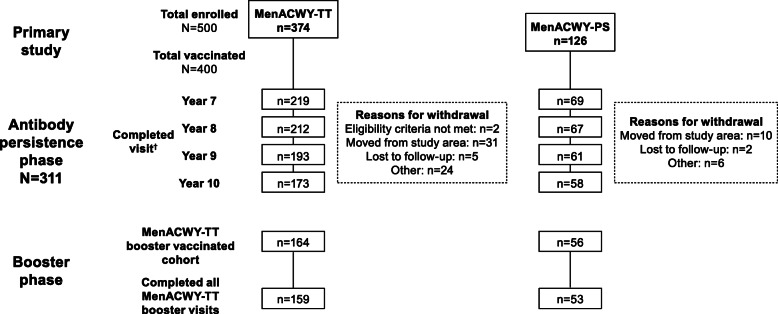
Subject disposition in the antibody persistence phase* and the booster phase.*MenACWY* meningococcal A, C, W, Y, *PS* polysaccharide, *TT* tetanus toxoid *Years 7–10 after the primary vaccination ^†^Completed all visits, *n* = 203

From the 400 subjects vaccinated in the primary study, 311 were enrolled in the antibody persistence phase (MenACWY-TT, *n* = 235; MenACWY-PS, *n* = 76). Overall, 219 (93.2%), 212 (90.2%), 193 (82.1%), and 173 (73.6%) subjects completed the Year 7, 8, 9, and 10 visit in the MenACWY-TT group, respectively; corresponding values in the MenACWY-PS group were 69 (90.8%), 67 (88.2%), 61 (80.3%), and 58 (76.3%) (Fig. 2). A total of 203 subjects (65.3%) completed all visits in the antibody persistence phase, with similar completion rates in both vaccine groups (MenACWY-TT, 64.7%; MenACWY-PS, 67.1%). Overall, 28 subjects (9.0%) missed at least 1 visit during the antibody persistence phase (MenACWY-TT, 8.9%; MenACWY-PS, 9.2%). At Year 10, 220 subjects (MenACWY-TT, *n* = 164; MenACWY-PS, *n* = 56) received a booster dose of MenACWY-TT. Approximately half of the subjects were male (52% and 55% in the antibody persistence phase and booster phase, respectively). The median age of enrollment was 22 and 24 years in the antibody persistence phase and booster phase, respectively (Table 1).

**Table 1:** Subject Demographics in the Antibody Persistence Phase and the MenACWY-TT Booster Phase

Demographic	Antibody Persistence Phase		MenACWY-TT Booster Phase	
	Primary MenACWY-TT	Primary MenACWY-PS	Primary MenACWY-TT	Primary MenACWY-PS
Total enrolled cohort, *n*	235	76	164	56
Sex, *n* (%)				
Male	127 (54.0)	36 (47.4)	93 (56.7)	29 (51.8)
Female	108 (46.0)	40 (52.6)	71 (43.3)	27 (48.2)
Age at enrollment, y				
Mean ± SD	25.3 ± 8.2	25.2 ± 8.4	26.8 ± 7.9	27.4 ± 8.7
Median (range)	22.0 (18–60)	22.0 (18–55)	24.0 (21–63)	24.5 (21–56)
Race, *n* (%)				
Asian/Southeast Asian	235 (100)	76 (100)	164 (100)	56 (100)

*MenACWY* meningococcal A, C, W, Y, *PS* polysaccharide, *SD* standard deviation, *TT* tetanus toxoid

The antibody persistence phase was for years 7–10 after the primary MenACWY-TT or MenACWY-PS vaccination

### Immunogenicity

#### Antibody persistence phase

**Fig. 3:**
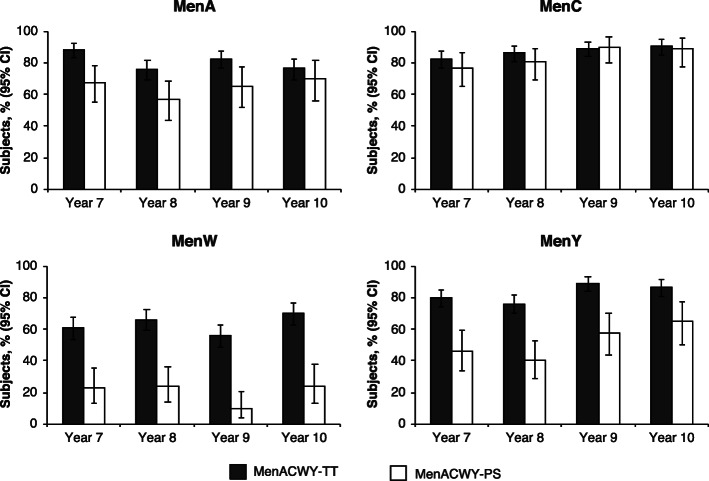
Subjects* with rSBA titers ≥1:8 at 7–10 years after the primary vaccination with MenACWY-TT or MenACWY-PS *MenACWY* meningococcal A, C, W, Y, *PS* polysaccharide, *rSBA* serum bactericidal antibody assay using baby rabbit complement, *TT* tetanus toxoid *In the according-to-protocol cohort for persistence

In subjects who received a primary vaccination of MenACWY-TT, rSBA titers were ≥1:8 across all 4 serogroups for 60.7% to 88.3% of subjects at Year 7, 66.2% to 86.3% at Year 8, 55.8% to 89.5% at Year 9, and 70.2% to 90.7% at Year 10 (Fig. 3). The percentages of subjects with rSBA titers ≥1:128 across all 4 serogroups at Years 7, 8, 9, and 10 were 52.4% to 76.7%, 60.6% to 70.9%, 50.5% to 86.3%, and 64.6% to 83.2%, respectively (Additional File 3: Table S2).

In subjects initially vaccinated with MenACWY-PS, rSBA titers were ≥ 1:8 across all 4 serogroups for 23.1% to 76.9% of subjects at Year 7, 23.9% to 80.6% at Year 8, 9.8% to 90.2% at Year 9, and 24.1% to 88.9% at Year 10 (Fig. 3). The percentages of subjects with rSBA titers ≥1:128 across all 4 serogroups were 16.9% to 61.5%, 16.4% to 64.2%, 9.8% to 67.2%, and 22.2% to 68.5%, respectively (Additional File 3: Table S2).

Descriptive comparisons suggested that the percentages of subjects achieving rSBA titers ≥1:8 were higher for MenACWY-TT than MenACWY-PS for serogroups A, W, and Y at most time points. The percentage of subjects in both groups achieving rSBA titers ≥1:8 was generally similar for serogroup C. The rSBA GMTs for serogroups A, W, and Y also were higher for MenACWY-TT than MenACWY-PS at most time points, but rSBA GMTs for serogroup C were higher for MenACWY-PS than MenACWY-TT (Additional File 3: Table S2). As >5% of subjects with serology results in the MenACWY-TT group at Year 7 and at Year 10 were excluded from the ATP cohort, analyses based on the total cohort at each year were also performed. Results based on the ATP or total cohorts were similar.

The percentages of subjects aged 11 to 17 years achieving rSBA titers ≥1:8 at Years 7 to 10 ranged from 56.6% to 91.2% in the MenACWY-TT group and 6.3% to 89.6% in the MenACWY-PS group across all 4 serogroups (Additional File 4: Table S3). The percentages of subjects aged 18 to 55 years achieving rSBA titers ≥1:8 at Years 7 to 10 ranged from 52.6% to 95.8% for MenACWY-TT recipients and 22.2% to 94.1% for MenACWY-PS recipients across all 4 serogroups (Additional File 4: Table S3).

#### Immunogenicity modeling

In both the MenACWY-TT and MenACWY-PS groups, observed rSBA GMTs during the persistence phase were similar to the estimated rSBA GMTs for all serogroups (Additional File 5: Figure S1).

#### Booster phase

The percentages of subjects meeting the definition of vaccine response at 1 month after the MenACWY-TT booster were 83.8% to 94.2% across all 4 serogroups for subjects in the MenACWY-TT group and 75.0% to 96.2% for subjects in the MenACWY-PS group (Table 2). In subjects aged 11 to 17 years, vaccine response across all 4 serogroups was seen in 86.5% to 97.0% of the MenACWY-TT group and 77.3% to 97.7% of the MenACWY-PS group. In subjects aged 18 to 55 years, vaccine response across the 4 serogroups was achieved by 66.7% to 86.4% of the MenACWY-TT group and 62.5% to 100% of the MenACWY-PS group. In all 4 serogroups, rSBA GMTs at 1 month after the MenACWY-TT booster dose were higher in the MenACWY-TT group compared with the MenACWY-PS group.

**Table 2:** Subjects With a Vaccine Response and rSBA GMTs After the MenACWY-TT Booster Dose

	Vaccine Response,^a^ % (CI)	GMT, % (CI)	
	1 Month After MenACWY-TT Booster	Before MenACWY-TT Booster	1 Month After MenACWY-TT Booster
Serogroup A			
Primary MenACWY-TT (*n* = 155)	87.7 (81.5, 92.5)	153.8 (108.1, 218.6)	4059.5 (3383.8, 4870.2)
Primary MenACWY-PS (*n* = 52)	88.5 (76.6, 95.6)	75.1 (41.4, 136.4)	3584.8 (2750.7, 4672.0)
Serogroup C			
Primary MenACWY-TT (*n* = 154)	90.9 (85.2, 94.9)	192.8 (140.6, 264.4)	13,823.5 (10,839.7, 17,628.7)
Primary MenACWY-PS (*n* = 52)	75.0 (61.1, 86.0)	212.4 (109.6, 411.8)	3444.3 (1998.5, 5936.0)
Serogroup W			
Primary MenACWY-TT (*n* = 154)	94.2 (89.2, 97.3)	166.2 (107.1, 257.9)	24,431.0 (17,351.4, 31,640.7)
Primary MenACWY-PS (*n* = 52)	96.2 (86.8, 99.5)	10.9 (6.1, 19.3)	5792.6 (3585.9, 9357.4)
Serogroup Y			
Primary MenACWY-TT (*n* = 154)	83.8 (77.0, 89.2)	363.7 (254.6, 519.4)	8958.4 (7601.6, 10,557.5)
Primary MenACWY-PS (*n* = 52)	92.3 (81.5, 97.9)	56.0 (28.8, 109.1)	5137.8 (3528.2, 7481.6)

*GMT* geometric mean titer, *MenACWY* meningococcal A, C, W, Y, *PS* polysaccharide, *rSBA* serum bactericidal antibody assay using baby rabbit complement, *TT* tetanus toxoid

Data are for subjects in the booster according-to-protocol cohort

^a^Vaccine response was defined as an rSBA titer ≥1:32 in subjects who were seronegative (rSBA titer <1:8) before booster vaccination and as a ≥4-fold increase from prevaccination titers in subjects who were seropositive before vaccination

One month after the booster MenACWY-TT vaccination, rSBA titers across all 4 serogroups were ≥1:8 for all subjects in the MenACWY-TT group and for 98.1% to 100% of subjects in the MenACWY-PS group (Additional File 6: Table S4). In both vaccine groups, the percentages of subjects with rSBA titers ≥1:8 at 1 month postbooster were ≥97.7% for subjects aged 11 to 17 years and ≥87.5% for those aged 18 to 55 years. The percentages of subjects with rSBA titers ≥1:128 were 100% for the MenACWY-TT and 96.2% to 100% for the MenACWY-PS groups, while the percentage was slightly higher for serogroup C in the MenACWY-TT group compared with the MenACWY-PS group (Additional File 6: Table S4).

### Safety

#### Local and general Reactogenicity events

**Fig. 4:**
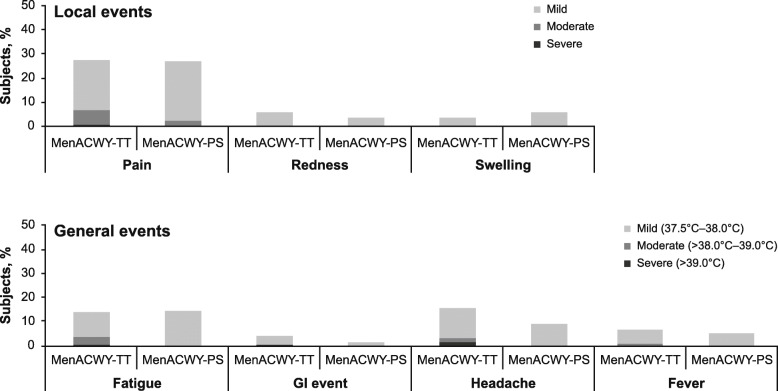
Subjects* reporting reactogenicity events within 4 days of receiving the MenACWY-TT booster. Both local and general reactogenicity events by intensity and the primary vaccine group are shown. Intensity scales are summarized in Additional File 1: Table S1. *GI* gastrointestinal, *MenACWY* meningococcal A, C, W, Y, *PS* polysaccharide, *TT* tetanus toxoid *In the booster total vaccinated cohort for safety

Local and general reactogenicity events occurring within 4 days after the MenACWY-TT booster were reported by 67 subjects (40.9%) and 21 subjects (37.5%) in the MenACWY-TT and MenACWY-PS groups, respectively. Local events (pain, redness, swelling) occurred in 49 subjects (29.9%) and 15 subjects (26.8%) in the MenACWY-TT and MenACWY-PS groups, respectively. General reactogenicity events (fatigue, fever, gastrointestinal event, headache) occurred in 40 subjects (24.4%) and 12 subjects (21.4%) in the MenACWY-TT and MenACWY-PS groups, respectively (Fig. 4; Additional File 7: Table S5). The investigators considered reactogenicity events to be related to the booster vaccination in 65 subjects (39.6%) and 21 subjects (37.5%) in the MenACWY-TT and MenACWY-PS groups, respectively.

The vast majority of reactogenicity events with MenACWY-TT booster dosing, excluding fever, were mild or moderate in intensity (97%). No subjects reported fever >39.0 °C after MenACWY-TT booster dosing. Four subjects (all in the MenACWY-TT group) reported a severe general reactogenicity event (fatigue in 1 subject, gastrointestinal event in 1 subject, headache in 2 subjects). One subject reported a severe local reactogenicity event (pain in a subject in the MenACWY-TT group).

#### Adverse events

Adverse events occurring within 31 days postbooster were reported in 15 subjects (9.1%) in the MenACWY-TT group and 2 subjects (3.6%) in the MenACWY-PS group. Of these AEs, 3 (1.8%) in the MenACWY-TT group and none in the MenACWY-PS group were considered by investigators as related to vaccination. The AEs considered related to the MenACWY-TT booster were dizziness, hypoesthesia, and oropharyngeal pain (*n* = 1 each). There were no reports of meningococcal disease or Guillain-Barré syndrome during the follow-up period for this analysis. Severe AEs were reported in 1 subject in the MenACWY-TT group (influenza-like illness) and in none of the subjects in the MenACWY-PS group. No NOCIs or SAEs were reported.

## Discussion

Protective immune responses to MenACWY-TT persisted in most subjects 10 years after the primary vaccination at age 11 to 55 years. Specifically, the percentages of subjects achieving protective rSBA titers for serogroups A, W, and Y were higher in the MenACWY-TT group than in the MenACWY-PS group and similar between the 2 groups for serogroup C for all time points. Together, these findings suggest the immunogenicity of MenACWY-TT vaccination in adolescents and adults (aged 11–55 years) against meningococcal disease may last up to 10 years postvaccination.

Limited data are available regarding the long-term persistence of antibody responses following the primary vaccination with meningococcal conjugate vaccines. Retrospective studies suggest that circulating antibodies after MenACWY-DT and MenACWY-CRM_197_ vaccination decrease within 3 to 8 years after dosing [5, 7]. In addition, data are available from follow-up of primary quadrivalent meningococcal conjugate vaccine studies regarding the persistence of antibody responses up to 5 years after a single dose in adolescents and young adults [11–16]. To the best of our knowledge, the current analysis of MenACWY-TT is the first to report long-term antibody persistence for a MenACWY conjugate vaccine for up to 10 years in subjects aged 11 to 55 years at the time of the primary vaccination.

A booster dose of MenACWY-TT administered 10 years after vaccination resulted in robust immunologic responses regardless of receipt of either the primary MenACWY-TT or MenACWY-PS vaccine. Reactogenicity events following a MenACWY-TT booster vaccination were mostly mild or moderate in intensity; the most common events were pain, fatigue, and headache. Results also showed the safety of MenACWY-TT, with 1.8% of AEs considered related to vaccination. No NOCIs or SAEs were reported after MenACWY-TT booster dosing. These data for MenACWY-TT booster dosing in individuals aged 11 to 55 years are consistent with data for the immunogenicity of the MenACWY-TT booster in other age groups, including children, adolescents, and young adults [13, 17–21].

The primary vaccination study included >300 adolescents aged 11 to 17 years who received either MenACWY-TT (*n* = 225) or MenACWY-PS (*n* = 76) [10]. Older adolescents and young adults are a critical population for meningococcal vaccination because they have the highest rates of meningococcal carriage and are therefore a reservoir for transmission; importantly, there is a secondary peak in meningococcal disease incidence in this age group [22, 23]. Thus, evidence of long-term antibody persistence up to 10 years following the primary MenACWY-TT vaccination in those aged 11 to 17 years suggests the protection afforded by vaccination extends throughout the second peak in meningococcal disease incidence and could reduce the high meningococcal carriage rates in this population.

The primary vaccination study also included nearly 200 adults aged 18 to 55 years [10]. In the current study, long-term persistence of antibody response to primary MenACWY-TT vaccination of up to 10 years and robust booster responses were observed in this population. These findings have potential implications regarding recommendations for MenACWY vaccination of adults, including those related to occupational risk and international travel.

Two strengths of this study are the long-term assessment of immunogenicity and safety for up to 10 years after the primary MenACWY-TT or MenACWY-PS vaccination and the high percentage of subjects returning for the long-term persistence evaluation. A limitation of the study is the small sample size. Moreover, the single study population from the Philippines may limit generalizing the results to other populations. Another limitation is that vaccine responses following a booster dose with MenACWY-PS among subjects who were initially vaccinated with MenACWY-TT were not investigated in this study. While it remains possible that subjects who receive a booster with MenACWY-PS could demonstrate strong initial immune responses following the booster dose, consistent with the high antibody responses seen after primary MenACWY-PS vaccination [10], the long-term antibody responses and GMTs with MenACWY-PS are not as robust as those achieved with MenACWY-TT [9]. Importantly, conjugate meningococcal vaccines elicit immunologic memory, providing extended periods of protection, while polysaccharide meningococcal vaccines do not [5]. Additionally, repeated dosing with polysaccharide meningococcal vaccines does not increase antibody titers. This suggests that use of a MenACWY-PS booster may not be an ideal strategy for providing long-term protection.

## Conclusions

MenACWY-TT administered as a single dose to adolescents and adults aged 11 to 55 years resulted in antibody responses persisting in most subjects up to 10 years after primary vaccination. In addition, a booster MenACWY-TT dose administered 10 years after the primary MenACWY-TT or MenACWY-PS vaccination elicited protective antibody titers and showed an acceptable safety and tolerability profile. These long-term antibody persistence results and booster dosing findings for MenACWY-TT in adolescents and adults provide important insights regarding long-term protection from the primary vaccination and the benefits of booster dosing, particularly for protection of adolescents who are at high risk of IMD and for adults who are at risk because of international travel or occupational exposure.

## Supplementary information

**Additional File 1: Table S1.** Intensity Scales for Local and General Reactogenicity Events. This table provides an overview of the intensity scales used to describe local and general reactogenicity events.

**Additional File 2: Supplementary Appendix.** Immunogenicity Modeling Methods. This document provides a summary of the approach used to model immunogenicity, including model fit and coding information.

**Additional File 3: Table S2.** Subjects* With rSBA Titers ≥1:8 and ≥1:128 and GMTs 7–10 Years After Primary Vaccination. This table displays antibody persistence data for primary vaccination with MenACWY-TT or MenACWY-PS at Years 7, 8, 9, and 10 after vaccination, including rSBA titers and rSBA GMTs.

**Additional File 4: Table S3.** Subjects* With rSBA Titers ≥1:8 7–10 Years After Primary Vaccination by Age Group. This table displays rSBA titers for primary vaccination with MenACWY-TT or MenACWY-PS at Years 7, 8, 9, and 10 after vaccination by age group.

**Additional File 5: Figure S1.** Observed and Estimated Year 10 rSBA GMTs* by Primary Vaccine for Each Meningococcal Serogroup. This figure compares observed rSBA GMTs in the persistence phase with modeling estimated data for each meningococcal serogroup.

**Additional File 6: Table S4.** Subjects* With rSBA Titers ≥1:8 and ≥1:128 Before and 1 Month After MenACWY-TT Booster Dose. This table displays immunogenicity data before and after a booster dose with MenACWY-TT for each meningococcal serogroup.

**Additional File 7: Table S5.** Subjects* Reporting Reactogenicity Events After MenACWY-TT Booster Dose by Intensity and Primary Vaccine Group. This table displays reactogenicity data after a booster dose with MenACWY-TT by primary vaccine and intensity of event.

## Data Availability

Upon request, and subject to certain criteria, conditions, and exceptions (see https://www.pfizer.com/science/clinical-trials/trial-data-and-results for more information), Pfizer will provide access to individual de-identified participant data from Pfizer-sponsored global interventional clinical studies conducted for medicines, vaccines, and medical devices (1) for indications that have been approved in the United States and/or European Union or (2) in programs that have been terminated (ie, development for all indications has been discontinued). Pfizer will also consider requests for the protocol, data dictionary, and statistical analysis plan. Data may be requested from Pfizer trials 24 months after study completion. The de-identified participant data will be made available to researchers whose proposals meet the research criteria and other conditions, and for which an exception does not apply, via a secure portal. To gain access, data requestors must enter into a data access agreement with Pfizer.

## Abbreviations

AE: Adverse event
ATP: According to protocol
DT: Diphtheria toxoid
GMT: Geometric mean titer
IMD: Invasive meningococcal disease
MenACWY: Meningococcal A, C, W, Y
NOCI: New onset of chronic illnesses
PS: Polysaccharide
SD: Standard deviation
TT: Tetanus toxoid
rSBA: serum bactericidal antibody assay using baby rabbit complement
SAE: Serious adverse event
