# Supplementary material for: A phase 2b/3b MenACWY-TT study of long-term antibody persistence after primary vaccination and immunogenicity and safety of a booster dose in individuals aged 11 through 55 years
Source: BMC Infect Dis. 2020 Jun 18;20:426. doi: 10.1186/s12879-020-05104-5 (PMC7301505; doi:10.1186/s12879-020-05104-5)
Supplement: Supplementary file 1 — Additional File 1: Table S1. Intensity Scales for Local and General Reactogenicity Events. This table provides an overview of the intensity scales used to describe local and general reactogenicity events. [file 12879_2020_5104_MOESM1_ESM.docx]

**Additional File 1: Table S1. Intensity Scales for Local and General Reactogenicity Events**

| **Event** | **Intensity** | **Definition** |
| --- | --- | --- |
| Pain | Mild | Any pain neither interfering with nor preventing  normal everyday activities |
|  | Moderate | Painful when limb is moved and interferes with everyday activities |
|  | Severe | Significant pain at rest; prevents normal everyday activities |
| Redness | Mild | >0–≤20 mm |
|  | Moderate | >20–≤50 mm |
|  | Severe | >50 mm |
| Swelling | Mild | >0–≤20 mm |
|  | Moderate | >20–≤50 mm |
|  | Severe | >50 mm |
| Fatigue | Mild | Easily tolerated |
|  | Moderate | Interferes with normal activity |
|  | Severe | Prevents normal activity |
| Gastrointestinal symptoms | Mild | Easily tolerated |
|  | Moderate | Interferes with normal activity |
|  | Severe | Prevents normal activity |
| Headache | Mild | Easily tolerated |
|  | Moderate | Interferes with normal activity |
|  | Severe | Prevents normal activity |
