# Supplementary material for: A phase 2b/3b MenACWY-TT study of long-term antibody persistence after primary vaccination and immunogenicity and safety of a booster dose in individuals aged 11 through 55 years
Source: BMC Infect Dis. 2020 Jun 18;20:426. doi: 10.1186/s12879-020-05104-5 (PMC7301505; doi:10.1186/s12879-020-05104-5)
Supplement: Supplementary file 2 — Additional File 2: Supplementary Appendix. Immunogenicity Modeling Methods. This document provides a summary of the approach used to model immunogenicity, including model fit and coding information. [file 12879_2020_5104_MOESM2_ESM.docx]

**Additional File 2: Supplementary Appendix**

**Immunogenicity Modeling Methods**

A longitudinal model considering vaccine group, age cohort, and all available immunogenicity time points up to the last time point at Year 10 was fitted. The primary purpose of the model was to evaluate selection bias, and time points were considered categorical.

Assay results for each time point were included in the model provided that those results were part of the according-to-protocol cohort for that time point. Results below the cutoffs for the serum bactericidal antibody assay using baby rabbit complement were set at half the value of the cutoff. The model was fitted via the proc mixed procedure according to the following code, where “age_cat” corresponded to the age group in the primary vaccination study:

*Model 1 – Repeated model on all available time points*

*PROC MIXED data=sero;*

*CLASS group time age_cat;*

*MODEL log_val = group | time age_cat;*

*Repeated time / TYPE=UN SUBJECT=pid;*

*Lsmeans group*time;*

*RUN;*
