# Supplementary material for: A phase 2b/3b MenACWY-TT study of long-term antibody persistence after primary vaccination and immunogenicity and safety of a booster dose in individuals aged 11 through 55 years
Source: BMC Infect Dis. 2020 Jun 18;20:426. doi: 10.1186/s12879-020-05104-5 (PMC7301505; doi:10.1186/s12879-020-05104-5)
Supplement: Supplementary file 3 — Additional File 3: Table S2. Subjects* With rSBA Titers ≥1:8 and ≥1:128 and GMTs 7–10 Years After Primary Vaccination. This table displays antibody persistence data for primary vaccination with MenACWY-TT or MenACWY-PS at Years 7, 8, 9, and 10 after vaccination, including rSBA titers and rSBA GMTs. [file 12879_2020_5104_MOESM3_ESM.docx]

## Additional File 3: Table S2. Subjects* With rSBA Titers ≥1:8 and ≥1:128 and GMTs 7–10 Years After Primary Vaccination

|  | **Year** | **n** | **Subjects With rSBA ≥1:8,**  **% (95% CI)** | **Subjects With rSBA ≥1:128,**  **% (95% CI)** | **rSBA GMT, % (95% CI)** |
| --- | --- | --- | --- | --- | --- |
| Serogroup A |  |  |  |  |  |
| MenACWY-TT | 7 | 206 | 88.3 (83.2, 92.4) | 73.8 (67.2, 79.7) | 220.8 (167.2, 291.5) |
|  | 8 | 208 | 76.0 (69.6, 81.6) | 60.6 (53.6, 67.3) | 104.8 (77.1, 142.4) |
|  | 9 | 190 | 82.6 (76.5, 87.7) | 75.3 (68.5, 81.2) | 227.8 (165.0, 314.5) |
|  | 10 | 162 | 76.5 (69.3, 82.8) | 67.9 (60.1, 75.0 | 142.5 (100.4, 202.1) |
| MenACWY-PS | 7 | 65 | 67.7 (54.9, 78.8) | 46.2 (33.7, 59.0) | 54.5 (31.1, 95.8) |
|  | 8 | 67 | 56.7 (44.0, 68.8) | 40.3 (28.5, 53.0) | 44.1 (24.3, 80.0) |
|  | 9 | 61 | 65.6 (52.3, 77.3) | 59.0 (45.7, 71.4) | 81.2 (44.2, 149.4) |
|  | 10 | 54 | 70.4 (56.4, 82.0) | 57.4 (43.2, 70.8) | 73.7 (40.9, 132.8) |
| Serogroup C |  |  |  |  |  |
| MenACWY-TT | 7 | 206 | 82.5 (76.6, 87.4) | 61.7 (54.6, 68.3) | 105.3 (79.7, 139.1) |
|  | 8 | 204 | 86.3 (80.8, 90.7) | 68.1 (61.3, 74.5) | 155.4 (118.3, 204.0) |
|  | 9 | 190 | 89.5 (84.2, 93.5) | 66.8 (59.7, 73.5) | 173.3 (129.9, 231.1) |
|  | 10 | 161 | 90.7 (85.1, 94.7) | 72.7 (65.1, 79.4) | 181.4 (134.6, 244.4) |
| MenACWY-PS | 7 | 65 | 76.9 (64.8, 86.5) | 61.5 (48.6, 73.3) | 156.7 (82.7, 297.1) |
|  | 8 | 67 | 80.6 (69.1, 89.2) | 64.2 (51.5, 75.5) | 240.6 (125.4, 461.8) |
|  | 9 | 61 | 90.2 (79.8, 96.3) | 67.2 (54.0, 78.7) | 264.9 (147.7, 474.9) |
|  | 10 | 54 | 88.9 (77.4, 95.8) | 68.5 (54.4, 80.5) | 234.0 (122.3, 447.0) |
| Serogroup W |  |  |  |  |  |
| MenACWY-TT | 7 | 206 | 60.7 (53.7, 67.4) | 52.4 (45.4, 59.4) | 83.2 (57.0, 121.5) |
|  | 8 | 207 | 66.2 (59.3, 72.6) | 60.9 (53.9, 67.6) | 119.7 (82.8, 173.1) |
|  | 9 | 190 | 55.8 (48.4, 63.0) | 50.5 (43.2, 57.8) | 71.7 (48.0, 107.0) |
|  | 10 | 161 | 70.2 (62.5, 77.1) | 64.6 (56.7, 72.0) | 161.5 (104.8, 248.9) |
| MenACWY-PS | 7 | 65 | 23.1 (13.5, 35.2) | 16.9 (8.8, 28.3) | 10.0 (6.3, 15.9) |
|  | 8 | 67 | 23.9 (14.3, 35.9) | 16.4 (8.5, 27.5) | 10.8 (6.7, 17.5) |
|  | 9 | 61 | 9.8 (3.7, 20.2) | 9.8 (3.7, 20.2) | 6.7 (4.5, 10.0) |
|  | 10 | 54 | 24.1 (13.5, 37.6) | 22.2 (12.0, 35.6) | 11.9 (6.8, 21.0) |
| Serogroup Y |  |  |  |  |  |
| MenACWY-TT | 7 | 206 | 80.1 (74.0, 85.3) | 76.7 (70.3, 82.3) | 270.2 (195.2, 373.8) |
|  | 8 | 206 | 76.2 (69.8, 81.9) | 70.9 (64.2, 77.0) | 181.6 (130.2, 253.3) |
|  | 9 | 190 | 89.5 (84.2, 93.5) | 86.3 (80.6, 90.9) | 460.6 (346.2, 612.7) |
|  | 10 | 161 | 87.0 (80.8, 91.7) | 83.2 (76.5, 88.6) | 387.0 (274.1, 546.4) |
| MenACWY-PS | 7 | 65 | 46.2 (33.7, 59.0) | 38.5 (26.7, 51.4) | 32.7 (17.8, 60.1) |
|  | 8 | 67 | 40.3 (28.5, 53.0) | 37.3 (25.8, 50.0) | 26.0 (14.5, 46.6) |
|  | 9 | 61 | 57.4 (44.1, 70.0) | 50.8 (37.7, 63.9) | 57.8 (30.6, 109.2) |
|  | 10 | 54 | 64.8 (50.6, 77.3) | 53.7 (39.6, 67.4) | 63.2 (33.4, 119.6) |

GMT=geometric mean titer; MenACWY=meningococcal A, C, W, Y; PS=polysaccharide; rSBA=serum bactericidal antibody assay using baby rabbit complement; TT=tetanus toxoid.

*In the according-to-protocol cohort for persistence.
