# Supplementary material for: A phase 2b/3b MenACWY-TT study of long-term antibody persistence after primary vaccination and immunogenicity and safety of a booster dose in individuals aged 11 through 55 years
Source: BMC Infect Dis. 2020 Jun 18;20:426. doi: 10.1186/s12879-020-05104-5 (PMC7301505; doi:10.1186/s12879-020-05104-5)
Supplement: Supplementary file 4 — Additional File 4: Table S3. Subjects* With rSBA Titers ≥1:8 7–10 Years After Primary Vaccination by Age Group. This table displays rSBA titers for primary vaccination with MenACWY-TT or MenACWY-PS at Years 7, 8, 9, and 10 after vaccination by age group. [file 12879_2020_5104_MOESM4_ESM.docx]

## Additional File 4: Table S3. Subjects* With rSBA Titers ≥1:8 at 7–10 Years After Primary Vaccination by Age Group

|  | **Age Group (years)** | **Year** | **n** | **Subjects With rSBA ≥1:8,**  **% (95% CI)** |
| --- | --- | --- | --- | --- |
| Serogroup A |  |  |  |  |
| MenACWY-TT | 11–17 | 7 | 145 | 90.3 (84.3, 94.6) |
|  |  | 8 | 154 | 76.6 (69.1, 83.1) |
|  |  | 9 | 152 | 84.9 (78.2, 90.2) |
|  |  | 10 | 137 | 78.8 (71.0, 85.3) |
|  | 18–55 | 7 | 61 | 83.6 (71.9, 91.8) |
|  |  | 8 | 54 | 74.1 (60.3, 85.0) |
|  |  | 9 | 38 | 73.7 (56.9, 86.6) |
|  |  | 10 | 25 | 64.0 (42.5, 82.0) |
| MenACWY-PS | 11–17 | 7 | 48 | 70.8 (55.9, 83.0) |
|  |  | 8 | 51 | 58.8 (44.2, 72.4) |
|  |  | 9 | 48 | 66.7 (51.6, 79.6) |
|  |  | 10 | 45 | 77.8 (62.9, 88.8) |
|  | 18–55 | 7 | 17 | 58.8 (32.9, 81.6) |
|  |  | 8 | 16 | 50.0 (24.7, 75.3) |
|  |  | 9 | 13 | 61.5 (31.6, 86.1) |
|  |  | 10 | 9 | 33.3 (7.5, 70.1) |
| Serogroup C |  |  |  |  |
| MenACWY-TT | 11–17 | 7 | 145 | 80.7 (73.3, 86.8) |
|  |  | 8 | 153 | 86.3 (79.8, 91.3) |
|  |  | 9 | 152 | 88.8 (82.7, 93.3) |
|  |  | 10 | 137 | 91.2 (85.2, 95.4) |
|  | 18–55 | 7 | 61 | 86.9 (75.8, 94.2) |
|  |  | 8 | 51 | 86.3 (73.7, 94.3) |
|  |  | 9 | 38 | 92.1 (78.6, 98.3) |
|  |  | 10 | 24 | 87.5 (67.6, 97.3) |
| MenACWY-PS | 11–17 | 7 | 48 | 70.8 (55.9, 83.0) |
|  |  | 8 | 51 | 76.5 (62.5, 87.2) |
|  |  | 9 | 48 | 89.6 (77.3, 96.5) |
|  |  | 10 | 45 | 88.9 (75.9, 96.3) |
|  | 18–55 | 7 | 17 | 94.1 (71.3, 99.9) |
|  |  | 8 | 16 | 93.8 (69.8, 99.8) |
|  |  | 9 | 13 | 92.3 (64.0, 99.8) |
|  |  | 10 | 9 | 88.9 (51.8, 99.7) |
| Serogroup W |  |  |  |  |
| MenACWY-TT | 11–17 | 7 | 145 | 62.1 (53.6, 70.0) |
|  |  | 8 | 154 | 66.2 (58.2, 73.6) |
|  |  | 9 | 152 | 56.6 (48.3, 64.6) |
|  |  | 10 | 137 | 69.3 (60.9, 76.9) |
|  | 18–55 | 7 | 61 | 57.4 (44.1, 70.0) |
|  |  | 8 | 53 | 66.0 (51.7, 78.5) |
|  |  | 9 | 38 | 52.6 (35.8, 69.0) |
|  |  | 10 | 24 | 75.0 (53.3, 90.2) |
| MenACWY-PS | 11–17 | 7 | 48 | 20.8 (10.5, 35.0) |
|  |  | 8 | 51 | 19.6 (9.8, 33.1) |
|  |  | 9 | 48 | 6.3 (1.3, 17.2) |
|  |  | 10 | 45 | 24.4 (12.9, 39.5) |
|  | 18–55 | 7 | 17 | 29.4 (10.3, 56.0) |
|  |  | 8 | 16 | 37.5 (15.2, 64.6) |
|  |  | 9 | 13 | 23.1 (5.0, 53.8) |
|  |  | 10 | 9 | 22.2 (2.8, 60.0) |
| Serogroup Y |  |  |  |  |
| MenACWY-TT | 11–17 | 7 | 145 | 77.9 (70.3, 84.4) |
|  |  | 8 | 154 | 77.9 (70.5, 84.2) |
|  |  | 9 | 152 | 89.5 (83.5, 93.9) |
|  |  | 10 | 137 | 85.4 (78.4, 90.8) |
|  | 18–55 | 7 | 61 | 85.2 (73.8, 93.0) |
|  |  | 8 | 52 | 71.2 (56.9, 82.9) |
|  |  | 9 | 38 | 89.5 (75.2, 97.1) |
|  |  | 10 | 24 | 95.8 (78.9, 99.9) |
| MenACWY-PS | 11–17 | 7 | 48 | 45.8 (31.4, 60.8) |
|  |  | 8 | 51 | 43.1 (29.3, 57.8) |
|  |  | 9 | 48 | 54.2 (39.2, 68.6) |
|  |  | 10 | 45 | 64.4 (48.8, 78.1) |
|  | 18–55 | 7 | 17 | 47.1 (23.0, 72.2) |
|  |  | 8 | 16 | 31.3 (11.0, 58.7) |
|  |  | 9 | 13 | 69.2 (38.6, 90.9) |
|  |  | 10 | 9 | 66.7 (29.9, 92.5) |

MenACWY=meningococcal A, C, W, Y; PS=polysaccharide; rSBA=serum bactericidal antibody assay using baby rabbit complement; TT=tetanus toxoid.

*In the according-to-protocol cohort for persistence.
