# Supplementary material for: A phase 2b/3b MenACWY-TT study of long-term antibody persistence after primary vaccination and immunogenicity and safety of a booster dose in individuals aged 11 through 55 years
Source: BMC Infect Dis. 2020 Jun 18;20:426. doi: 10.1186/s12879-020-05104-5 (PMC7301505; doi:10.1186/s12879-020-05104-5)
Supplement: Supplementary file 5 — Additional File 5: Figure S1. Observed and Estimated Year 10 rSBA GMTs* by Primary Vaccine for Each Meningococcal Serogroup. This figure compares observed rSBA GMTs in the persistence phase with modeling estimated data for each meningococcal serogroup. [file 12879_2020_5104_MOESM5_ESM.docx]

## Additional File 5: Figure S2. Observed and Estimated Year 10 rSBA GMTs* by Primary Vaccine for Each Meningococcal Serogroup


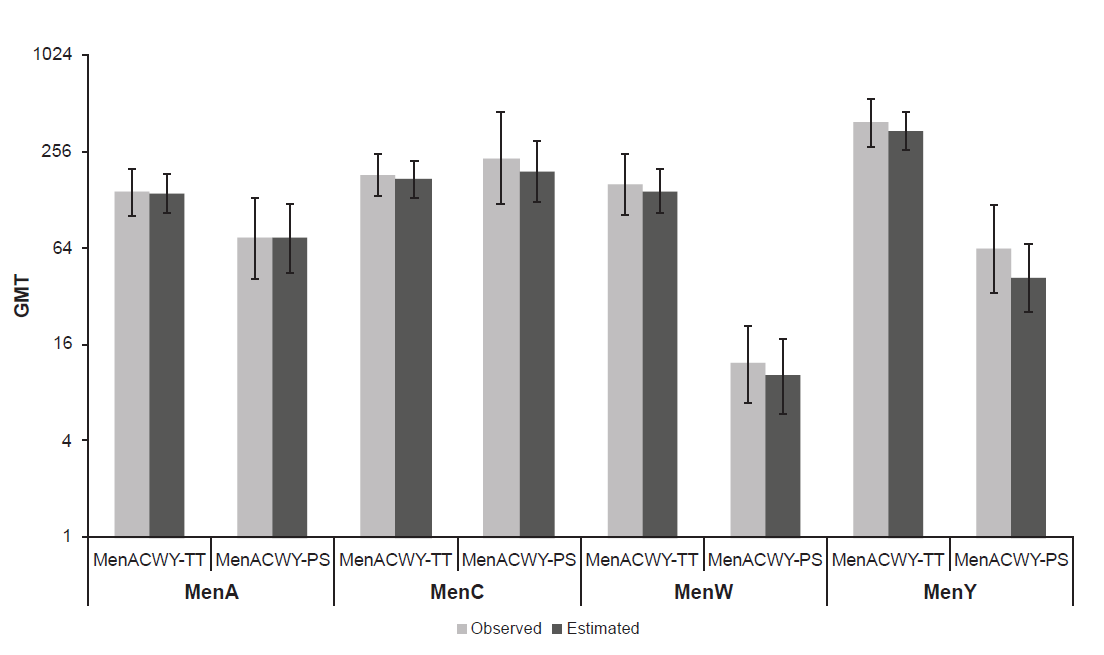


Modeling accounts for subjects who withdrew from the study and supports that the observed results are robust. GMT=geometric mean titer; MenACWY=meningococcal A, C, W, Y; PS=polysaccharide; rSBA=serum bactericidal antibody assay using baby rabbit complement; TT=tetanus toxoid.

*In the according-to-protocol cohort for persistence.
