# Supplementary material for: A phase 2b/3b MenACWY-TT study of long-term antibody persistence after primary vaccination and immunogenicity and safety of a booster dose in individuals aged 11 through 55 years
Source: BMC Infect Dis. 2020 Jun 18;20:426. doi: 10.1186/s12879-020-05104-5 (PMC7301505; doi:10.1186/s12879-020-05104-5)
Supplement: Supplementary file 6 — Additional File 6: Table S4. Subjects* With rSBA Titers ≥1:8 and ≥1:128 Before and 1 Month After MenACWY-TT Booster Dose. This table displays immunogenicity data before and after a booster dose with MenACWY-TT for each meningococcal serogroup. [file 12879_2020_5104_MOESM6_ESM.docx]

**Additional File 6: Table S4. Subjects* With rSBA Titers ≥1:8 and ≥1:128 Before and 1 Month After MenACWY-TT Booster Dose**

|  | **Subjects With rSBA ≥1:8, % (95% CI)** | | **Subjects With rSBA ≥1:128, % (95% CI)** | |
| --- | --- | --- | --- | --- |
|  | **Before MenACWY-TT Booster** | **1 Month After  MenACWY-TT Booster** | **Before MenACWY-TT Booster** | **1 Month After  MenACWY-TT Booster** |
| Serogroup A |  |  |  |  |
| Primary MenACWY-TT (n=155) | 78.1 (70.7, 84.3) | 100 (97.6, 100) | 69.7 (61.8, 76.8) | 100 (97.6, 100) |
| Primary MenACWY-PS (n=52) | 71.2 (56.9, 82.9) | 100 (93.2, 100) | 57.7 (43.2, 71.3) | 100 (93.2, 100) |
| Serogroup C |  |  |  |  |
| Primary MenACWY-TT (n=154–155) | 90.9 (85.2, 94.9) | 100 (97.6, 100) | 72.7 (65.0, 79.6) | 100 (97.6, 100) |
| Primary MenACWY-PS (n=52) | 88.5 (76.6, 95.6) | 98.1 (89.7, 100) | 67.3 (52.9, 79.7) | 96.2 (86.8, 99.5) |
| Serogroup W |  |  |  |  |
| Primary MenACWY-TT (n=154–155) | 71.4 (63.6, 78.4) | 100 (97.6, 100) | 64.9 (56.8, 72.4) | 100 (97.6, 100) |
| Primary MenACWY-PS (n=52) | 21.2 (11.1, 34.7) | 98.1 (89.7, 100) | 19.2 (9.6, 32.5) | 98.1 (89.7, 100) |
| Serogroup Y |  |  |  |  |
| Primary MenACWY-TT (n=154–155) | 86.4 (79.9, 91.4) | 100 (97.6, 100) | 82.5 (75.5, 88.1) | 100 (97.6, 100) |
| Primary MenACWY-PS (n=52) | 61.5 (47.0, 74.7) | 100 (93.2, 100) | 50.0 (35.8, 64.2) | 100 (93.2, 100) |

MenACWY=meningococcal A, C, W, Y; PS=polysaccharide; rSBA=serum bactericidal antibody assay using baby rabbit complement; TT=tetanus toxoid.

*In the booster according-to-protocol cohort for immunogenicity.
