# Supplementary material for: A phase 2b/3b MenACWY-TT study of long-term antibody persistence after primary vaccination and immunogenicity and safety of a booster dose in individuals aged 11 through 55 years
Source: BMC Infect Dis. 2020 Jun 18;20:426. doi: 10.1186/s12879-020-05104-5 (PMC7301505; doi:10.1186/s12879-020-05104-5)
Supplement: Supplementary file 7 — Additional File 7: Table S5. Subjects* Reporting Reactogenicity Events After MenACWY-TT Booster Dose by Intensity and Primary Vaccine Group. This table displays reactogenicity data after a booster dose with MenACWY-TT by primary vaccine and intensity of event. [file 12879_2020_5104_MOESM7_ESM.docx]

**Additional File 7: Table S5. Subjects* Reporting Reactogenicity Events After MenACWY-TT Booster Dose by Intensity and Primary Vaccine Group**

|  | **Primary MenACWY-TT Vaccination**  **(n=159)** | **Primary MenACWY-PS Vaccination**  **(n=53)** |
| --- | --- | --- |
| **Local events, n (%)** | | |
| Pain | 43 (27.0) | 14 (26.4) |
| Mild | 33 (20.8) | 13 (24.5) |
| Moderate | 9 (5.7) | 1 (1.9) |
| Severe | 1 (0.6) | 0 |
| Redness | 9 (5.7) | 2 (3.8) |
| Mild | 9 (5.7) | 2 (3.8) |
| Moderate | 0 | 0 |
| Severe | 0 | 0 |
| Swelling | 6 (3.8) | 3 (5.7) |
| Mild | 6 (3.8) | 3 (5.7) |
| Moderate | 0 | 0 |
| Severe | 0 | 0 |
| **General events, n (%)** | | |
| Fatigue | 23 (14.5) | 8 (15.1) |
| Mild | 17 (10.7) | 8 (15.1) |
| Moderate | 5 (3.1) | 0 |
| Severe | 1 (0.6) | 0 |
| Related | 20 (12.6) | 8 (15.1) |
| Gastrointestinal event | 7 (4.4) | 1 (1.9) |
| Mild | 6 (3.8) | 1 (1.9) |
| Moderate | 0 (0.0) | 0 |
| Severe | 1 (0.6) | 0 |
| Related | 6 (3.8) | 1 (1.9) |
| Headache | 25 (15.7) | 5 (9.4) |
| Mild | 20 (12.6) | 5 (9.4) |
| Moderate | 3 (1.9) | 0 |
| Severe | 2 (1.3) | 0 |
| Related | 22 (13.8) | 5 (9.4) |
| Fever | 11 (6.9) | 3 (5.7) |
| 37.5°C–38.0°C | 10 (6.3) | 3 (5.7) |
| >38.0°C–39.0°C | 1 (0.6) | 0 |
| >39.0°C | 0 | 0 |
| Related | 11 (6.9) | 3 (5.7) |

MenACWY: meningococcal A, C, W, Y; PS=polysaccharide; TT=tetanus toxoid.

Intensity scales are summarized in **Additional File 1: Table S1**.

*In the booster total vaccinated cohort for safety.
